# Cumulative effect of cotrimoxazole, isoniazid and opportunistic infection prophylaxis on CD4 response among people living with HIV on first-line ART in Ari Zone, Southern Ethiopia

**DOI:** 10.1186/s12981-026-00873-6

**Published:** 2026-03-23

**Authors:** Sitotaw Ahmed, Tegenu Tento, Nurhussen Ahmed

**Affiliations:** 1 Department of Statistics, Jinka University, Jinka, Ethiopia; 2 Department of Surgery, Yekatite 12 Hospital Medical College, Addis Abeba, Ethiopia

**Keywords:** Adherence, Cumulative, Prophylactic, Synergistic, People living with HIV

## Abstract

**Background:**

Prophylactic medications are commonly used to prevent tuberculosis and opportunistic infections in people living with HIV (PLWH) who are on first-line antiretroviral therapy (ART). However, their cumulative influence on cluster of differentiation 4 (CD4) responses is still a subject of ongoing investigation.

**Methods:**

A retrospective chart review was conducted utilizing secondary data derived from medical records of 500 people living with HIV (≥ 15 years) who initiated first-line ART at Jinka General Hospital in Ari Zone, South Ethiopia, between September 2019 and January 2025. CD4 response was defined as an increase of ≥ 50 cells/µL from baseline to the most recent measurement. The most recent CD4 measurement taken up to January 2025 was used for analysis. The primary outcome measure was a binary variable for CD4 response, defined as an increase of ≥ 50 cells/µL from baseline to end-line counts. Multivariable binary logistic regression was used to assess independent and interaction effects of prophylactic therapies on CD4 response.

**Results:**

Among 500 participants, 50.2% demonstrated an improvement in CD4 count. Use of cotrimoxazole preventive therapy (CPT), isoniazid preventive therapy (IPT), and opportunistic infection (OI) medications was each independently associated with higher odds of CD4 improvement. However, interaction analysis showed that the CPT-OI combination and the triple CPT-IPT-OI regimen showed a statistically significant interaction suggesting a possible synergistic association with CD4 recovery beyond their individual additive effects. Predicted probabilities of CD4 improvement increased progressively with dual and triple prophylactic combinations compared with no prophylaxis.

**Conclusion:**

The combined use of prophylactic medications was significantly associated with CD4 recovery in people living with HIV on first-line ART. These findings emphasize the importance of integrated preventative strategies, particularly in resource-limited settings, and highlight the need for adherence support among individuals with limited formal education and those living in rural areas. The study’s generalizability is limited, however, due to its single-center retrospective design and the use of a broad category for “OI prophylaxis” without specifying individual drugs.

**Supplementary Information:**

The online version contains supplementary material available at 10.1186/s12981-026-00873-6.

## Introduction

The death rate of human immunodeficiency virus (HIV) has been high since 2003, even though antiretroviral therapy (ART) was introduced in Ethiopia early [1]. Newer medications have significantly increased the effectiveness and safety of ART [2]. People living with HIV are recommended to receive prophylaxis. To prevent tuberculosis (TB) and opportunistic infections, prophylactic therapies like cotrimoxazole preventive therapy (CPT), isoniazid preventive therapy (IPT), and other opportunistic infection (OI) prophylaxis are often used [3–6]. While these therapies are effective in reducing infection-related morbidity and mortality, their combined use may introduce drug–drug interactions and cumulative effects that could influence immunological recovery, particularly the cluster of differentiation 4 (CD4) cell count response [7]. In Ari Zone, Southern Ethiopia, treatment outcomes among people living with HIV (PLWH) are further shaped by socioeconomic constraints and health system limitations, which may affect access to prophylaxis and adherence.

The most important laboratory indicator of immune function in PLWH is the CD4 count [8–10]. Previous studies have identified factors such as age, sex, baseline CD4 count, adherence, and co-infections as predictors of CD4 recovery [11–14]. However, although prophylactic medicines have been shown to be effective in preventing certain infections, evidence on the cumulative and interactive effects of multiple prophylactic therapies on CD4 response remains limited. It remains unclear whether the simultaneous use of these therapies produces antagonistic effects (lower CD4 response) or synergistic effects (improved immunological recovery).

The study aimed to evaluate the cumulative impact of prophylactic therapies on CD4 cell count response using interaction terms among adult PLWH receiving first-line ART at Jinka General Hospital, with a focus on determining whether combined prophylaxis produces synergistic or antagonistic effects on immune recovery.

## Methods

### Study design and setting

A retrospective chart review was conducted using secondary data derived from ART clinic registers and participants’ medical records at Jinka General Hospital, covering the period from September 2019 to January 2025. The most recent CD4 measurement available up to January 2025 was used for analysis.

### Study population and sampling

The study included PLWH aged ≥ 15 years who initiated first-line ART and had documented baseline and end-line CD4 measurements. A simple random sample of 500 participants was selected.

### Study variables

The outcome variable was CD4 response, defined as a binary variable:

1 = if CD4 count increased by ≥ 50 cells/µL from baseline (improved).

0 = CD4 count increased by < 50 cells/µL (not improved).

Independent factors included age, gender, marital status, residence, functional level, education, world health organization (WHO) clinical stage, tuberculosis status, infections, comorbidities, haemoglobin level, body mass index (BMI), use of OI prophylaxis, CPT, IPT, ART regimen, adherence level, and interaction terms among prophylactic therapies.

### Operational definitions

CD4 Response: Increase of ≥ 50 cells/µL from baseline to end-line CD4 counts.

Cumulative burden of prophylaxis: Concurrent use of two or more prophylactic therapies (CPT, IPT, and/or OI prophylaxis).

Interaction effect: The combined effect of multiple prophylactic therapies on CD4 response.

### Statistical analysis

The binary logistic regression and chi-square tests were performed to explore relationships between explanatory variables and the binary outcome [15, 16]. Model selection was performed using a stepwise procedure based on the Akaike Information Criterion (AIC). Model adequacy was evaluated using the Hosmer-Lemeshow goodness of fit test, variance inflation factors (VIF) for multicollinearity, and the area under the curve (AUC) with receiver operating characteristic curve (ROC) for model discrimination [17].

## Results

### Descriptive statistics and association

According to the output in Table 1, 64.4% of 500 PLWH were female and 72.8% lived in urban areas. In 50.2% (*n* = 251) cases, CD4 counts increased by ≥ 50 cells/µL.

### Analytical results

#### Results of binary logistic regression model without interaction term

In the initial multivariable regression model with no interaction terms, urban residence was associated with higher odds of CD4 recovery (OR = 1.84, *p* = 0.006). Educational attainment was also significant, with higher odds observed among participants with primary education (OR = 2.31, *p* = 0.001) and secondary or higher education (OR = 1.95, *p* = 0.013) compared with those with no formal education. Use of OI prophylaxis (OR = 2.70, *p* = 0.003) and normal haemoglobin levels (OR = 12.02, *p* = 0.021) were also associated with CD4 improvement.

#### Results of binary logistic regression analysis after interaction term

The major objective of the study was to establish the cumulative effect of the prophylactic medications. For this reason, interaction terms were added in the multivariable model. As shown in Table 2 several predictors remained significant at the 5% level of significance, including residence, education status, haemoglobin status, and the usage of OI medications, CPT, and IPT. The model also identified statistically significant interaction terms for CPT × OI and IPT × CPT × OI.

**Fig. 1:**
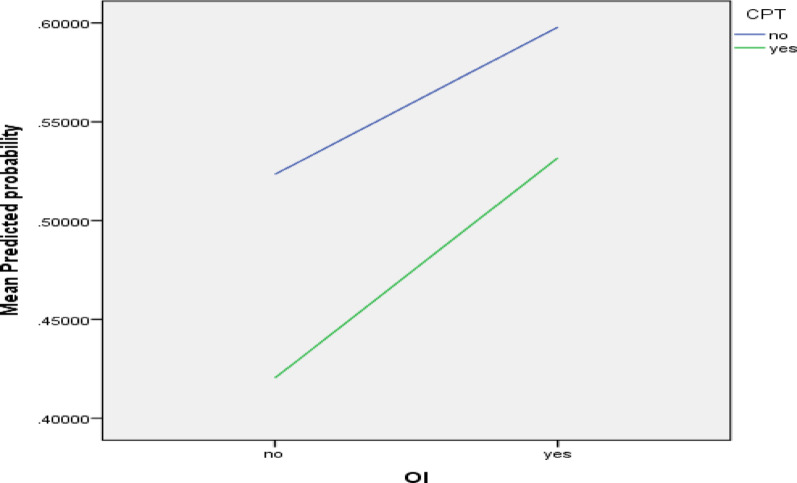
Interaction plot for CPT and OI prophylaxis on CD4 immune recoveryInteraction plot shows non-parallel trends for CPT and OI prophylaxis groups. It is consistent with a significant interaction effect. *CPT* cotrimoxazole preventive therapy, *OI prophylaxis* opportunistic infection prophylaxis

The interaction plot (Fig. 1) depicts the predicted probabilities of CD4 improvement for CPT and OI prophylaxis groups.

Predicted probabilities were computed for each prophylaxis combination. The baseline group (without prophylaxis) had an estimated probability of 43%. Individual prophylaxis types showed higher predicted probabilities. The predicted values increased further for dual (CPT + OI) and triple combos (IPT + CPT + OI medicines).

#### Assessment of goodness of fit of the model

After fitting the logistic regression model for categorical outcomes, model adequacy, fit, and predictive usefulness were evaluated. Pearson’s Chi-square, likelihood ratio tests (LRT), the Hosmer and Lemeshow goodness of fit test, and the ROC curve are some of the procedures that were used in this study to evaluate the goodness of fit.

The omnibus likelihood ratio test yielded significant results (χ² = 199.97, df = 40, *p* < 0.001). Pseudo-R² values (Cox & Snell = 0.35; Nagelkerke = 0.47) indicated that predictors explained 35–47% of variability in CD4 recovery. The Hosmer-Lemeshow yielded an accepting model fit (χ² = 8.69, df = 8, *p* = 0.370).

**Fig. 2:**
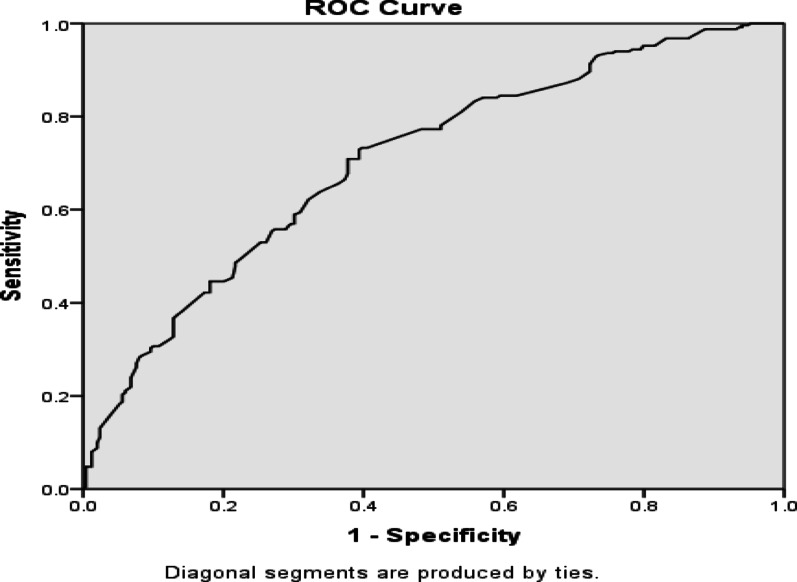
Receiver operating characteristic (ROC) curve for the logistic regression model predicting CD4 responseThe ROC curve illustrates the discriminative ability of the fitted model. *ROC* receiver operating characteristic, *AUC* area under the curve

Classification accuracy was 72.2% with sensitivity 81.3% and specificity 63.2%. The ROC curve in Fig. 2 showed an AUC of 0.706.

### Discussion

The goal of this study was to assess the cumulative effect of preventive therapies on CD4 response in PLWH receiving first-line ART at Jinka General Hospital in Ari Zone, Southern Ethiopia. The results shed light on how these preventive interventions, both individually and collectively, affect immunological recovery as measured by CD4 count changes.

The multivariable logistic regression results without interaction terms demonstrated that urban residence, higher education, use of OI medications, and normal haemoglobin were all significant predictors of CD4 level improvement. Participants living in urban areas had higher odds of CD4 recovery than rural residents; this could be attributed to improved access to healthcare facilities in urban areas, such as rapid laboratory testing, prophylactic availability, and adherence support. Similarly, participants with secondary or higher education were more likely to experience immunological recovery, suggesting that improved health literacy may support better adherence to ART and preventive regimens. People living with HIV who received OI prophylaxis and had normal haemoglobin levels were more likely to experience CD4 recovery. This finding highlights the need for routine anaemia screening and management, and the clinical value of addressing opportunistic infections as part of comprehensive HIV care. These findings were consistent with earlier studies that linked socioeconomic factors and anaemia treatment with improved immunological outcomes [8, 18, 19].

When interaction terms were included in the logistic regression model, the potential synergistic effect of combination prophylaxis became apparent. The interaction of CPT and OI medications (CPT_OI) and the three-way interaction (IPT_CPT_OI) were significantly associated with improved CD4 counts, beyond additive effects, with progressively higher predicted probabilities of immunological recovery. The triple combination was associated with the highest predicted probability of CD4 improvement, suggesting a possible statistical interaction between preventive therapies that should be interpreted cautiously. This complements previous data that comprehensive prophylactic regimes boost immunological recovery [7, 3].

The study’s findings suggest the potential benefit of combination prophylaxis in rural communities with limited resources and access to health care. This suggests that prophylaxis regimens are more than an add-on, and emphasizing adherence to preventive regimens, particularly among rural and individuals with limited formal education, because it could play an important role alongside ART, although further prospective studies are needed to confirm their impact on treatment outcomes. Continuous monitoring is required to balance the advantages against potential drug-drug interactions. The model displayed adequate fit and classification performance, confirming the reliability of these findings.

This study yielded several important findings, but they should be taken in light of the study’s inherent limitations. The first limitation is that the analysis was carried out at a single location (Jinka General Hospital) which may limit its generalizability to other settings with differing individual profiles, health care system capacities and patterns of prophylactic usage. The second limitation relates to the retrospective study design. It is based on routinely recorded data, which may contain missing information, documentation discrepancies, or unmeasured confounders such as timing of prophylactic commencement, duration of medication, or drug supply disruption. The third drawback is that the study employed a broad category of “OI prophylaxis” without defining the specific drugs used. Different OI medications can have varying effects, so putting them together may mask key distinctions. Fourth, some interaction estimates yielded large odds ratios with wide confidence intervals, likely reflecting sparse data in certain prophylaxis combinations, therefore, these estimates should be interpreted cautiously. Fifth, adherence level, although a well-established predictor of antiretroviral therapy outcomes, was not statistically significant in this study. This outcome could reflect the limitations of typical adherence recording techniques, probable misclassification, or a lack of objective adherence monitoring. In addition to the above limitations, the temporal relationship between initiation of prophylactic therapies and the timing of CD4 measurement could not be fully established. Accordingly, well-designed multicentre or prospective studies with complete documentation of specific prophylactic medicines, timing, and duration, as well as more reliable adherence measuring methods, are necessary.

## Conclusion

The combination of IPT, CPT, and OI medicines was significantly associated with improved CD4 cell count recovery. This suggests a possible synergistic favourable effect on CD4 cell count recovery among PLWH receiving first-line ART. Urban location, higher education level, and normal haemoglobin levels were also linked to improved immunological recovery.

These findings support the potential importance of integrated preventive regimens (IPT, CPT, and OI medications) as part of comprehensive HIV care, although causal effects cannot be inferred from this study. Prioritizing prophylactic adherence support programs, especially for rural residents and those with limited formal education, is also crucial.

**Table 1:** Binary comparisons of socio-demographic and clinical characteristics between people living with HIV (PLWH) who did and did not achieve satisfactory immune reconstitution

Variable	Category	CD4 response		P-value
		Improved	Not improved	
		n (%)	n (%)	
Sex	Male	86(48.3)	92(51.7)	0.531
	Female	165(65.7)	157(63.1)	
Residence	Urban	168(46.2)	196(53.8)	**0.003**
	Rural	83(61.0)	53(39.0)	
Education status	No	122(57.3)	90(42.5)	**0.002**
	Primary	85(49.7)	86(50.3)	
	Secondary and above	44(37.6)	73(62.4)	
Marital status	Single	19(52.8)	17(47.2)	0.736
	Married	160(49.1)	166(50.9)	
	Divorced	52(50.0)	52(50.0)	
	Widowed	20(58.8)	14(41.2)	
Functional status	Working	177(52.4)	161(47.6)	**0.216**
	Ambulatory	18(39.1)	28(60.9)	
	Bedridden	56(48.3)	60(51.7)	
Infections	Yes	103(46.0)	121(54.0)	**0.089**
	No	148(53.6)	128(46.4)	
Comorbidity	Yes	83(48.3)	89(51.7)	0.529
	No	168(51.2)	160(48.8)	
Clinical stage	Stage I	116(57.4)	86(42.6)	**0.067**
	Stage II	77(44.8)	95(55.2)	
	Stage III	51(46.4)	59(53.6)	
	Stage IV	7(43.8)	9(56.2)	
Drug regimen	TDF/3TC/EFV	2(50.0)	2(50.0)	**0.086**
	TDF/3TC/DTG	239(51.5)	225(48.5)	
	Other	10(31.2)	22(68.8)	
OI prophylaxis	Yes	141(61.0)	90(39.0)	**0.000**
	No	110(40.9)	159(59.1)	
CPT	Yes	122(43.3)	160(56.7)	**0.000**
	No	129(59.2)	89(40.8)	
IPT	Yes	239(49.6)	243(50.4)	**0.155**
	No	12(66.7)	6(33.3)	
Adherence status	Good	149(50.3)	147(49.7)	0.845
	Fair	38(52.8)	34(47.2)	
	Poor	64(48.5)	68(51.5)	
Age category	< 25	28(42.4)	38(57.6)	0.277
	25–34	106(50.5)	104(49.5)	
	35–44	86(50.6)	84(49.4)	
	45–54	18(50.0)	18(50.0)	
	>=55	13(72.2)	5(27.8)	
BMI category	Underweight	39(55.7)	31(44.3)	0.673
	Normal	169(48.6)	179(51.4)	
	Overweight	35(51.5)	33(48.5)	
	Obese	8(57.1)	6(42.9)	
Haemoglobin cat.	Anaemic	11(91.7)	1(8.3)	**0.004**
	Normal	240(49.2)	248(50.8)	

Values are presented as n (column %), where percentages represent the proportion of participants within each CD4 response category that achieved or did not achieve CD4 improvement and n is size of the group. Chi-square test was used for comparisons. Significant predictors of CD4 improvement (*p* < 0.25) are highlighted in bold

*PLWH* people living with HIV, *CPT* Cotrimoxazole preventive therapy, *IPT* Isoniazid preventive therapy, *OI prophylaxis* Opportunistic infection prophylaxis, *BMI* Body mass index

**Table 2:** Binary logistic regression results with interaction terms for predictors of CD4 improvement among people living with HIV (PLWH) on antiretroviral therapy

Predictor	B	S.E.	Wald		Df		*p*-value	Exp(B)	95% CI for Exp(B)	
Residence (Urban)	0.581	0.225	6.646		1		0.010	1.788	1.150–2.782	
Education: (Primary)	0.846	0.259	10.628		1		0.001	2.330	1.401–3.875	
Education: (secondary & above)	0.632	0.275	5.286		1		0.021	1.881	1.098–3.224	
OI prophylaxis (Yes)	1.152	0.550	4.385		1		0.036	3.166	1.077–9.309	
CPT (Yes)	2.359	1.081	4.765		1		0.029	10.585	1.272–88.044	
IPT (Yes)	3.333	1.287	6.711		1		0.010	28.029	2.251–348.999	
Haemoglobin (Normal)	2.398	1.076	4.966		1		0.026	10.999	1.335–90.627	
CPT × OI interaction	1.691	0.647		6.832		1	0.009	5.425		1.526–19.278
IPT × CPT × OI interaction	3.550	1.309		7.351		1	0.007	34.827		2.676–451.184

Reference categories are rural, no education, anaemic haemoglobin, and not taking prophylaxis

*CI* confidence interval, *CPT* cotrimoxazole preventive therapy, *IPT* isoniazid preventive therapy, *OI* prophylaxis: opportunistic infection prophylaxis

## Supplementary Information

Supplementary Material 1.

## Data Availability

The datasets created and analysed during the current investigation are made available upon reasonable request from the authors.

## Abbreviations

AIC: Akaike Information Criterion
ART: Antiretroviral Therapy
AUC: Area Under the Curve
BMI: Body Mass Index
CD4: Cluster of Differentiation 4
CPT: Cotrimoxazole Preventive Therapy
HIV: Human Immunodeficiency Virus
IPT: Isoniazid Preventive Therapy
LRT: Likelihood Ratio Test
OI: Opportunistic Infection
PLWH: People Living With HIV
ROC: Receiver Operating Characteristics
VIF: Variance Inflation Factor
WHO: World Health Organization
