# Supplementary material for: Cumulative effect of cotrimoxazole, isoniazid and opportunistic infection prophylaxis on CD4 response among people living with HIV on first-line ART in Ari Zone, Southern Ethiopia
Source: AIDS Res Ther. 2026 Mar 23;23:55. doi: 10.1186/s12981-026-00873-6 (PMC13130529; doi:10.1186/s12981-026-00873-6)
Supplement: Supplementary file 1 — Supplementary Material 1. [file 12981_2026_873_MOESM1_ESM.docx]

| Variable |  | B | S.E. | Wald |  | Df |  | Sig. |  | Exp(B) | 95% C.I. Lower | 95% C.I. Upper |
| --- | --- | --- | --- | --- | --- | --- | --- | --- | --- | --- | --- | --- |
| Residence(Urban) |  | 0.610 | 0.220 | 7.713 |  | 1 |  | 0.005 |  | 1.840 | 1.197 | 2.831 |
| Educstatus(Primary) |  | 0.837 | 0.254 | 10.840 |  | 1 |  | 0.001 |  | 2.310 | 1.403 | 3.802 |
| Educstatus(Secondary and above) |  | 0.668 | 0.268 | 6.224 |  | 1 |  | 0.013 |  | 1.949 | 1.154 | 3.294 |
| OI(Yes) |  | 0.992 | 0.330 | 9.006 |  | 1 |  | 0.003 |  | 2.696 | 1.411 | 5.152 |
| CPT(Yes) |  | 0.054 | 0.346 | 0.024 |  | 1 |  | 0.877 |  | 1.055 | 0.535 | 2.081 |
| INH(Yes) |  | 0.757 | 0.547 | 1.913 |  | 1 |  | 0.167 |  | 2.131 | 0.729 | 6.227 |
| HemogCat(Normal) |  | 2.487 | 1.073 | 5.366 |  | 1 |  | 0.021 |  | 12.020 | 1.466 | 98.540 |
| Constant |  | -0.077 | 0.028 | 7.563 |  | 1 |  | 0.038 |  | .926 |  |  |

Table 1: Results of binary logistic regression model fit for selected variables without interaction term.

Interpretation of logistic regression coefficients

A logistic model was fitted to the data to test the research hypothesis regarding the relationship between the likelihood that CD4 level is related with the predictor variables. Result displayed in **Table 1** revealed that residence, education status, OI drugs, and Haemoglobin level were found to be significantly associated with CD4 level.

Patients living in urban areas had 1.840 higher odds of CD4 improvement compared to rural residents (p = 0.005), and those with primary education (OR = 2.310, p = 0.001) and secondary and above (OR = 1.949, p = 0.013) had significantly better immune recovery than patients with no education, highlighting the role of access to health services and awareness. Patients who had taken OI drugs had about 69.6% increased odds of CD4 improvement (OR = 2.696, p = 0.003), and also normal patients had 12.02 times higher odds of immune recovery compared to those with anaemic haemoglobin (p = 0.021).

Model diagnosis

Table 2: Overall model evaluation using likelihood ratio test

| **Model Summary** | | | |
| --- | --- | --- | --- |
| Step | -2 Log likelihood | Cox & Snell R Square | Nagelkerke R Square |
| 1 | 443.235^a^ | .350 | .467 |

The most common assessment of overall model fit in logistic regression is the likelihood ratio test, which is simply the chi-square difference between the null model (i.e., with the constant only) and the model containing the predictors. Under Model Summary we see that the -2 Log Likelihood statistics is 443.235. This statistic measures how poorly the model predicts the patient’s CD4 level improvement, the smaller the statistic the better the model. The Cox and Snell or Nagelkerke R^2^ is an analogous statistic in logistic regression to the coefficient of determination R^2^ in linear regression, but not close analogy. The model summary provides some approximation of R^2^ statistic in logistic regression. Cox and Snell’s R^2^ attempts to imitate multiple R^2^ based on likelihood. The result of Cox and Snell R^2^ indicates that 35% of the variation in the dependent variable is explained by the predictor variable (**Table 2**).

Based on the results in **Table 3**, the null hypothesis that there is no difference between the model with only a constant and the model with independent variables was rejected.

Table 3: Omnibus tests of model coefficients

| **Omnibus Tests of Model Coefficients** | | | | |
| --- | --- | --- | --- | --- |
|  | | Chi-square | Df | Sig. |
| Step 1 | Step | 199.971 | 40 | .000 |
|  | Block | 199.971 | 40 | .000 |
|  | Model | 199.971 | 40 | .000 |

The Hosmer-Lemeshow goodness-of-fit test is found to be not significant (x^2^ = 8.689, d.f = 8, p-value = 0.370). Thus, we do not have an evidence to reject the null hypothesis that the model fitted the data well.

Table 4**:** Hosmer-Lemeshow goodness-of-fit test

| **Hosmer and Lemeshow Test** | | | |
| --- | --- | --- | --- |
| Step | Chi-square | Df | Sig. |
| 1 | 8.686 | 8 | .370 |

Validation of predicted probabilities
